# Effects of Acetaminophen on Oxidant and Irritant Respiratory Tract Responses to Environmental Tobacco Smoke in Female Mice

**DOI:** 10.1289/ehp.1509851

**Published:** 2015-10-09

**Authors:** Gregory J. Smith, Joseph A. Cichocki, Bennett J. Doughty, Jose E. Manautou, Sven-Eric Jordt, John B. Morris

**Affiliations:** 1 Department of Pharmaceutical Sciences, School of Pharmacy, University of Connecticut, Storrs, Connecticut, USA; 2 Department of Anesthesiology, Duke University School of Medicine, Durham, North Carolina, USA

## Abstract

**Background:**

Although it is known that acetaminophen causes oxidative injury in the liver, it is not known whether it causes oxidative stress in the respiratory tract. If so, this widely used analgesic may potentiate the adverse effects of oxidant air pollutants.

**Objectives:**

The goal of this study was to determine if acetaminophen induces respiratory tract oxidative stress and/or potentiates the oxidative stress and irritant responses to an inhaled oxidant: environmental tobacco smoke (ETS).

**Methods:**

Acetaminophen [100 mg/kg intraperitoneal (ip)] and/or sidestream tobacco smoke (as a surrogate for ETS, 5 mg/m3 for 10 min) were administered to female C57Bl/6J mice, and airway oxidative stress was assessed by loss of tissue antioxidants [estimated by nonprotein sulfhydryl (NPSH) levels] and/or induction of oxidant stress response genes. In addition, the effects of acetaminophen on airway irritation reflex responses to ETS were examined by plethysmography.

**Results:**

Acetaminophen diminished NPSH in nasal, thoracic extrapulmonary, and lung tissues; it also induced the oxidant stress response genes glutamate-cysteine ligase, catalytic subunit, and NAD(P)H dehydrogenase, quinone 1, in these sites. ETS produced a similar response. The response to acetaminophen plus ETS was equal to or greater than the sum of the responses to either agent alone. Although it had no effect by itself, acetaminophen greatly increased the reflex irritant response to ETS.

**Conclusions:**

At supratherapeutic levels, acetaminophen induced oxidative stress throughout the respiratory tract and appeared to potentiate some responses to environmentally relevant ETS exposure in female C57Bl/6J mice. These results highlight the potential for this widely used drug to modulate responsiveness to oxidant air pollutants.

**Citation:**

Smith GJ, Cichocki JA, Doughty BJ, Manautou JE, Jordt SE, Morris JB. 2016. Effects of acetaminophen on oxidant and irritant respiratory tract responses to environmental tobacco smoke in female mice. Environ Health Perspect 124:642–650; http://dx.doi.org/10.1289/ehp.1509851

## Introduction

Oxidative stress results from an imbalance between antioxidants and prooxidants within a cell. Oxidative stress is a common mechanism for respiratory tract injury by inhaled as well as by systemically delivered toxic agents. For example, oxidative stress contributes to airway injury produced by inhaled diesel exhaust, ozone, and environmental tobacco smoke (ETS) (Holguin 2013). Systemically delivered toxicants such as styrene and naphthalene can be bioactivated throughout the respiratory tract and induce oxidative stress as well (Cruzan et al. 2012; Spiess et al. 2010). Oxidative stress contributes to the development and/or exacerbation of respiratory diseases, including asthma. For example, biomarkers of oxidative stress are elevated in asthma, and individuals with low antioxidant levels are susceptible to the development of this disease (Larkin et al. 2015; Riedl and Nel 2008). Additionally, oxidant air pollutants, including the ubiquitous indoor air pollutant ETS, are associated with increased prevalence and/or severity of asthma [Gilmour et al. 2006; Institute of Medicine (U.S.) Committee on the Assessment of Asthma and Indoor Air (IOM) 2000; Kanchongkittiphon et al. 2015]. This study focused on the potential for the over-the-counter analgesic acetaminophen (APAP, *N*-acetyl-*para*-aminophenol) to induce airway oxidative stress and potentiate the airway response to the inhaled oxidant stressor, ETS.

APAP is a commonly used medicine for relieving pain and reducing fever in adults and children, and it is a known hepatotoxicant. The majority of APAP is metabolized in the liver by glucuronidation and sulfation pathways and is safely excreted; however, a fraction of APAP is metabolically activated in the liver to the prooxidant metabolite *N*-acetyl-*p*-benzoquinone-imine (NAPQI) (McGill and Jaeschke 2013). NAPQI is highly reactive, causes cellular oxidative stress, and covalently binds to cellular macromolecules (Jaeschke et al. 2012). Detoxification of NAPQI consumes the important antioxidant glutathione (GSH). NAPQI induces the nuclear factor-erythroid 2-related factor-2 (NRF2)–dependent oxidative stress gene response pathway (Bataille and Manautou 2012; Klaassen and Reisman 2010), causing induction of genes involved in multiple detoxification pathways. One of the induced genes expresses the enzyme that catalyzes the rate-limiting step of GSH synthesis, glutamate-cysteine ligase, catalytic subunit (GCLC); another is NAD(P)H dehydrogenase, quinone 1 (NQO1), which is involved in the detoxification of reactive quinones (Aleksunes et al. 2006; Chan and Kwong 2000). Induction of NRF2 pathway genes can be used as a sensitive biomarker for cellular oxidative stress (Cichocki et al. 2014a, 2014b; Klaassen and Reisman 2010). Specifically, in the typical hierarchical response pattern to oxidants, induction of NRF2 genes occurs at doses lower than those needed to induce inflammation or to cause cytotoxicity (Nel et al. 2006).

The metabolic activation of APAP to NAPQI is catalyzed by a variety of cytochrome P450 isoforms including CYP2E1, CYP3A4, and CYP1A2 (Hinson et al. 2010). These CYPs are expressed in the respiratory tract, suggesting that similar metabolic activation may occur in this site as well (Ding and Kaminsky 2003). When administered directly to the lungs via intratracheal instillation, NAPQI induces a neurogenic inflammatory response (Nassini et al. 2010), and hepatotoxic doses of acetaminophen are known to deplete GSH and cause injury in the nose and lungs when administered systemically (Hart et al. 1995; Gu et al. 2005). It is not known whether the bioactivation capacity of respiratory tissues is sufficient to induce oxidative stress at nonhepatotoxic, supratherapeutic doses of APAP. Were APAP to induce oxidative stress in respiratory tissues, it could enhance the response to other oxidant stressors. The present study was focused on determining whether APAP induces oxidative stress in respiratory tissues, and if so, whether APAP enhances the oxidative stress and respiratory tract irritant responses to environmentally relevant ETS exposure. ETS was selected because it is a common air pollutant and because both acetaminophen and ETS have been associated with increased prevalence of asthma (Etminan et al. 2009; Gilmour et al. 2006; IOM 2000; McBride 2011).

The hypothesis that APAP acts as a prooxidant in the airways and enhances the response to ETS was tested in a mouse model by determining whether APAP and/or APAP + ETS *a*) cause a loss of GSH, and *b*) activate the oxidant stress response pathway, as indicated by the activation of two NRF2-dependent genes: *Gclc* and *Nqo1*. Because the intent was to determine if the dosages of APAP or ETS were sufficient to alter normal homeostatic levels of nonprotein sulfhydryl (NPSH) or gene expression, the data for these parameters were expressed as a percentage of the control. Precise quantitative comparisons between treatment groups were made within individual experiments (which shared the same control group). Only generalized comparisons were made across experiments where control levels may have differed. The effects of APAP on the response to ETS were further characterized by examining the ETS-induced irritation reflex response. This response is caused by ETS stimulation of nasal trigeminal chemosensory nerves through the oxidant-sensitive transient receptor potential ankyrin 1 (TRPA1) channel (Andrè et al. 2008).

## Materials and Methods

*Experimental approaches*. The first studies were aimed at determining whether APAP induced oxidative stress in respiratory tissues. To this end, animals were euthanized 0–3 hr after intraperitoneal (ip) APAP administration, and nasal respiratory/transitional mucosa (RTM), intrathoracic airways [tracheal/mainstream bronchial mucosa (TBM)], lung (left lobe), and liver samples were collected. Mice were euthanized by anesthetization with urethane (1.3 g/kg) followed by exsanguination in the laboratory between 1000 and 1200 hours. Oxidative stress was assessed by determination of tissue nonprotein sulfhydryl (NPSH, as a surrogate for GSH) levels and by the expression of two NRF2-dependent oxidant stress response genes: *Gclc* and *Nqo1* (Cichocki et al. 2014b). The response of *Nrf2* ^–/–^ mice to APAP was also investigated to confirm a role for NRF2 in any gene expression changes. Plasma levels of acetaminophen were determined in animals euthanized 15 min after 100 mg/kg dosing [the expected time of peak plasma concentration based on previous studies (Gu et al. 2005; Lin et al. 1996)] to assess the therapeutic relevancy of the APAP dosing regimen.

The interaction between APAP and ETS was assessed by examining the nasal RTM response of C57Bl/6J wild type mice (tissue NPSH levels and *Gclc* and *Nqo1* expression) to these agents alone and in combination. Sidestream smoke was used as a surrogate for ETS. Mice were exposed for 10 min to a nominal exposure concentration of 5 mg/m^3^ to approximate the ETS concentrations achieved in a closed automobile containing a smoker, or they were exposed to clean filtered air in the same apparatus (Sendzik et al. 2009). The 10-min duration corresponded to the burn time of a single cigarette. The effects of APAP on the reflex irritation response of mice to ETS were also examined. Stimulation of trigeminal chemosensory nerves causes a brainstem-mediated characteristic change in breathing pattern that can be assessed noninvasively during exposure as described in detail below (Alarie 1973; Vijayaraghavan et al. 1993; Willis et al. 2011). Stimulation of these nerves is also proinflammatory (Andrè et al. 2008; Caceres et al. 2009; Nassini et al. 2010). The rationale for the focus on the nose for the APAP-ETS study was multifold. The nose is the first airway that is exposed to ETS in the mouse, and it is a common site of inhaled toxicant-induced injury in the rodent (Morris 2012). Nasal injury in nose-breathing rodents is a sentinel for lower airway injury in mouth-breathing humans (Morris 2012; Cichocki et al. 2014b), and the ETS-induced irritant reflex response is mediated via the trigeminal nerve and, therefore, originates in the nose (Gloede et al. 2011; Morris 2012).

We examined the role of oxidative stress in inducing the irritant reflex response using multiple approaches. First, the effects of APAP on responses to the prooxidant irritant acrolein and the nonoxidant irritant cyclohexanone were examined to confirm whether any effects of APAP were oxidant-specific rather than generalized in nature. Cyclohexanone activates chemosensory nerves by the transient receptor potential vanillin 1 (TRPV1) receptor, and acrolein acts through TRPA1 (Bautista et al. 2006; Saunders et al. 2013). Second, the effects of APAP were examined in animals pretreated with 5-phenyl-1-pentyne (5-PP) to inhibit CYP metabolism (Morris 2013; Roberts et al. 1998) and bioactivation of APAP. Third, the GSH-depleting agent diethyl maleate (DEM) was administered to determine whether modulation of nasal GSH status could replicate the effects of APAP. This agent is conjugated with GSH via glutathione *S*-transferases, resulting in decreased tissue GSH levels (Boyland and Chasseaud 1967; Phimister et al. 2004).

*Animal procedures.* Female C57Bl/6J mice (9–11 weeks of age) were used for all experiments. Female mice were used because there is a rich database of respiratory reflex responses to irritants, including ETS, in female mice (Ha et al. 2015; Willis et al. 2011) and because female mice are more sensitive than male mice to the acute respiratory tract effects of metabolically activated toxicants (Van Winkle et al. 2002). Mice were obtained from Jackson Laboratories. Age-matched *Nrf2*-null and wild type (C57BL/6J background) mice were used. Initial *Nrf2* ^–/–^ breeding pairs were obtained from A. Slitt, University of Rhode Island. Mice were housed in American Association for Accreditation of Laboratory Animal Care–accredited facilities at the University of Connecticut under standard environmental conditions (12-hr light–dark cycle at 23°C). Mice were housed over hardwood shavings in groups of 5 mice per cage (Sani-Chip® Dry, P. J. Murphy Forest Products). Food (LabDiet®; PMI Nutrition International) and tap water were provided *ad libitum*. A total of 570 mice were used for these studies. All animals were treated humanely and with regard for alleviation of suffering. Animal procedures were approved by the University of Connecticut Institutional Animal Care and Use Committee.

Unless otherwise indicated, all chemicals were obtained from Sigma Aldrich. APAP, dissolved in 37°C saline (10 mg/mL), was administered via ip injection at doses of 60, 100, or 200 mg/kg. The cytochrome P450 inhibitor 5-PP (GFS Chemicals) was administered ip at a dose of 100 mg/kg (10 mg/mL in olive oil) 1 hr prior to APAP treatment where indicated by the experimental protocol (Morris 2013). DEM was administered at a dose of 250 mg/kg [0.33 M solution in olive oil, ip (Phimister et al. 2004)]. Control animals received injections of vehicle. Mice were exposed to airborne irritants as described below; irritant exposure concentrations were selected to produce demonstrable but submaximal irritation. Nasal RTM tissues were removed from the ventral portions of the nasal cavity by microdissection (Cichocki et al. 2014a). (Olfactory mucosa was not collected because this is neural, nonrespiratory, tissue.) The intrathoracic TBM airways and the left lobe of the lungs were removed. For NPSH determination, tissue samples were homogenized in 5% trichloroacetic acid–3 mM ethylenediaminetetraacetic acid (EDTA) and were stored at –80°C until analysis. For quantitative reverse transcriptase polymerase chain reaction (qRT-PCR), tissues were immediately placed in an aqueous RNA stabilization buffer, which contained saturating ammonium sulfate, 20 mM EDTA, and 25 mM sodium citrate, at pH 5.2 and were stored at –80°C until analysis.

*Breathing pattern analysis.* Mice were held in a double plethysmograph (Buxco Inc.) connected to a directed airflow nose-only inhalation chamber (CH Technologies) for irritant exposure to allow monitoring of breathing parameters during exposure. Animals were placed in the plethysmograph for a 15-min acclimatization period, a 5-min baseline period, and then a 10-min exposure to irritant. The sensory irritation reflex response, characterized by a pause (termed braking) at the onset of each expiration caused by glottal closure, was quantitated by measuring the duration of braking (Willis et al. 2011). Breathing patterns were monitored using a Buxco mouse pneumotachograph with a Buxco pressure transducer coupled to automated Iox 2 software (emka TECHNOLOGIES S.A.S). This software automatically measured the duration of braking for each breath and averaged those data over each minute (typically 150–300 breaths) to provide minute-by-minute averages over a 30-min measurement period. Plethysmographic-based assessment of breathing patterns based on 1-min averages is the long-accepted approach for assessment of sensory irritation (Alarie 1981; Vijayaraghavan et al. 1993; Morris et al. 2003).

*Respiratory irritant exposures.* Mice were exposed to ETS, acrolein, or cyclohexanone for 10 min. Control mice were exposed to clean filtered air in the same exposure chamber. Mice were continuously exposed to constant levels of irritant to allow for the most precise estimation of irritant- or APAP-induced changes in breathing. During exposure, clean or irritant-laden air was drawn into the headspace of the double plethysmograph at a flow rate of 1 L/min.

Acrolein (nominal concentration 2.5 ppm) atmospheres were generated by flash evaporation, and cyclohexanone (nominal concentration, 1,500 ppm) atmospheres were generated by passing filtered air through liquid cyclohexanone in a gas washing bottle. Airborne vapor concentrations were monitored by gas chromatography using a Varian 3800 gas chromatograph as described previously (Willis et al. 2011). To achieve constant-concentration smoke exposures, sidestream cigarette smoke was continuously generated by passing filtered and humidified air over a lit cigarette centered in one port of a 2-L four-neck boiling flask (Kimble-Chase). Highly concentrated smoke from the flask was drawn into a second identical flask by a peristaltic pump and was fed into the inhalation chamber using positive pressure. Smoke was generated from Kentucky 2R4F reference cigarettes (University of Kentucky) that had been stored for at least 24 hr at 55% relative humidity. Particulate levels for the smoke exposures were measured using a Casella Microdust Pro Analyzer (Casella CEL, Inc.). The nominal suspended particle concentration over the duration of the smoke exposures was 5 mg/m^3^. Airborne carbon monoxide (CO) levels were monitored continuously during exposure with a Digital CO Detector (DCO1001, General Tools). CO levels averaged 25–35 ppm throughout the exposures.

*Analytical techniques.* NPSH levels were used as a surrogate for GSH. NPSH was determined colorimetrically in a 96-well plate assay using a reduced GSH standard curve based on the method of Sedlak and Lindsay (1968). NPSH data were normalized to protein content using a colorimetric 96-well plate assay with a BSA standard curve based on the Lowry method (Lowry et al. 1951). NPSH data are expressed as a percentage of the control. Control NPSH levels were determined empirically, and the data for exposed groups are expressed as the average value among concurrent controls. Overall, control values averaged 18, 8, and 12 nmol/mg protein in RTM, TBM, and lung, respectively. To determine plasma APAP levels, blood was collected by cardiac puncture with heparinized syringes from mice euthanized 15 min after a 100-mg/kg dose of APAP. Plasma, prepared by centrifugation, was diluted 1:10 in 5% trichloroacetic acid–3 mM EDTA and then analyzed for APAP content by high-performance liquid chromatography (HPLC) based on the method of Lin et al. (1996). APAP eluted with a retention time of 11 min when using a mobile phase of 7% acetonitrile–0.1% trichloroacetic acid, a flow rate of 1 mL/min, and an Agilent Eclipse-Pro Plus LC-18 (5 μm, 4.6 × 250 mm) column, and was detected by UV absorbance at a wavelength of 254 nm. Levels were determined in plasma from 4 APAP-injected and 4 vehicle-injected mice (to confirm the absence of contaminating peaks).

For qRT-PCR of selected oxidant stress–response genes, total RNA was isolated from respiratory tissue homogenates using an RNeasy kit from Qiagen. One microgram of total RNA was used for first-strand cDNA synthesis by an iScript cDNA synthesis kit (Bio-Rad Laboratories, Inc.). RT-PCR was performed using SYBR Green as an indicator with an ABI 7500 Real-Time PCR system on the fast setting. PCR reactions contained 10 ng of cDNA (4 μL), 500 nmol of each primer (1 μL total), and 5 μL of 2x SYBR Green PCR Master mix for a total volume of 10 μL. PCR was performed according to the manufacturer’s recommended thermal cycling protocol. Data were normalized to β-actin as the internal reference control mRNA. Results are represented as the fold change in expression of target genes over control values calculated using the 2^–ΔΔCT^ method (Livak and Schmittgen 2001). Primers were designed with Life Technologies OligoPerfect™ designer and obtained from Invitrogen (Life Technologies). See Table 1 for a list of primer sequences.

**Table 1:** Mouse primer sequences for qRT-PCR (listed 5’-3’):

Gene	Forward	Reverse
*ActB*	GCAACGAGCGGTTCCG	CCCAAGAAGGAAGGCTGGA
*Nqo1*	TTTAGGGTCGTCTTGGCAAC	GTCTTCTCTGAATGGGCCAG
Gclc	TTCATGATCGAAGGACACCA	CTGCACATCTACCACGCAGT

*Statistical analysis.* Numbers of animals per group were selected to detect a 25% difference between groups based on our previous experience with the methodologies with α = 0.05 at 80% power. Data were analyzed using XLSTAT v.2011.2.06 (Addinsoft). Individual data values were excluded *a priori* if they deviated from the mean by more than three standard deviations. (Of the approximately 600 mice used in this study, data from 5 mice were excluded because of this exclusion criterion.) Data are reported as the mean ± SE unless otherwise indicated. Data were compared by analysis of variance (ANOVA) followed by the Newman–Keuls test as appropriate. Where appropriate, data were log_10_ transformed to correct for heteroscedasticity. Sensory irritation time-course data were analyzed by repeated-measures ANOVA followed by the Newman–Keuls test. A *p*-value < 0.05 was required for statistical significance.

## Results

**Figure 1:**
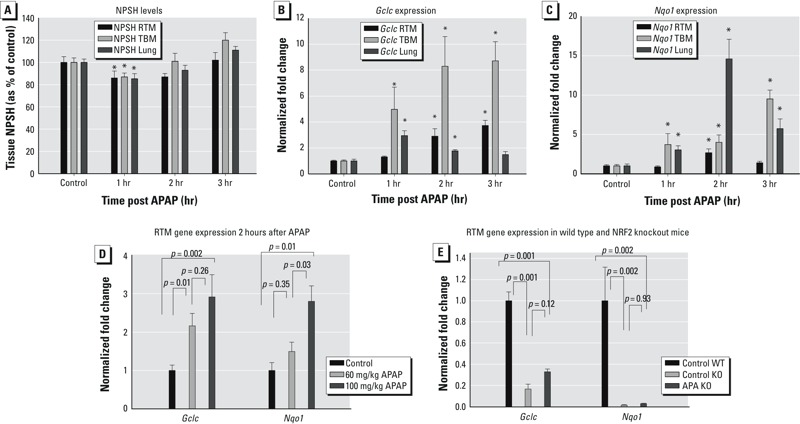
Nonprotein sulfhydryl (NPSH) levels and oxidant stress response gene expression in the respiratory tract following acetaminophen (APAP) administration. (*A*) Time course of NPSH levels in nasal respiratory/transitional mucosa (RTM), thoracic extrapulmonary airway mucosa [trachea/mainstem bronchi (TBM)], and lung parenchyma (Lung) 1, 2, or 3 hr after APAP administration [100 mg/kg, intraperitoneal (ip)]. Data are presented as the mean ± SE and are normalized to total protein. Groups contained 4–6 mice. (*B*,*C*) Time courses of (*B*) *Gclc* and (*C*) *Nqo1* expression in nasal RTM, TBM, and Lung 1, 2, or 3 hr after APAP administration (100 mg/kg, i.p). Data are presented as the mean fold change ± SE and are normalized to control values. Groups contained 4–6 mice. (*D*) Dose–response relationships for *Gclc* and *Nqo1* expression 2 hr after APAP administration (60, 100 mg/kg, ip). (*E*) Effects of APAP on *Gclc* and *Nqo1* expression in NRF2 wild type (WT) and knockout (KO) mice. Data are presented as the mean fold change + SE and are normalized to control values (*p*-values are indicated in the figure). Groups contained 4–6 mice. *p < 0.05 compared with the respective control.

*Airway oxidative stress responses to APAP.* To examine the time course of the response to APAP, mice were euthanized 1, 2, or 3 hr after treatment with 100 mg/kg APAP, and tissues were collected from RTM, TBM, and lung. In all tissues, NPSH levels were approximately 80% of control values 1 hr after treatment (*p* < 0.05) and returned to control levels by 2–3 hr after treatment (Figure 1A). At a dose of 60 mg/kg, RTM NPSH was not diminished by APAP, averaging 96 ± 7.7% of control values. Liver NPSH levels averaged 60 ± 3.9%, 83 ± 2.7%, and 106 ± 3.5%, at 1, 2, and 3 hr, respectively, after a 100-mg/kg dose of APAP. Both *Gclc* and *Nqo1* were significantly induced in all respiratory tissues at a dose of 100 mg/kg, albeit with somewhat differing magnitudes and time courses (Figure 1B,C). Dose–response relationships for RTM gene induction are shown in Figure 1D. Although *Gclc* was significantly induced at a dose of 60 mg/kg APAP, *Nqo1* was not. Both *Gclc* and *Nqo1* were significantly induced at a dose of 100 mg/kg APAP. In *Nrf2* ^–/–^ mice, basal RTM expression of *Gclc* and *Nqo1* were approximately 17% and 2% of wild type control, respectively (7- and 70-fold lower than wild type control, respectively) (Figure 1E). In APAP-treated mice, *Gclc* expression averaged 33% of wild type-control and *Nqo1* expression averaged 4% of control. Thus, 2-fold or lower induction of either gene was observed. The gene expression in APAP-treated knockout mice did not differ from that in control knockout mice. Serum APAP levels were determined in mice euthanized 15 min after a 100-mg/kg dose. No APAP (or contaminating peak) was detected in vehicle-injected controls (*n* = 4); APAP levels averaged 35 ± 6 μg/mL (*n* = 4) in treated mice.

**Figure 2:**
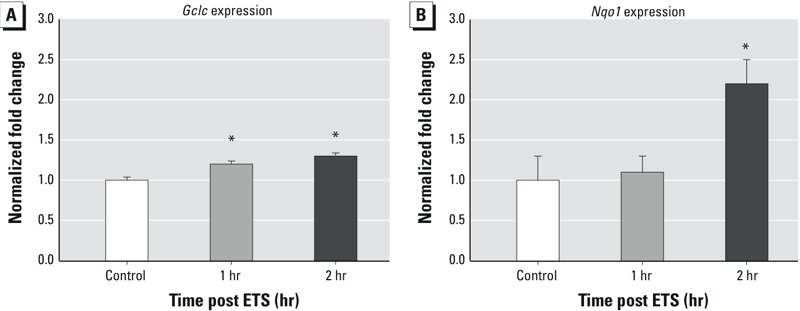
Time course of oxidant stress response gene expression in nasal respiratory/transitional mucosa following 10-min exposure to 6 mg/m^3^ environmental tobacco smoke (ETS). *n *= 6 in all groups. (*A*) *Gclc* and (*B*) *Nqo1* expression data are presented as mean fold change ± SE, and are normalized to control values. *p < 0.05 compared with respective control.

*APAP-ETS interaction.* Initial studies focused on the time course of NRF2-dependent gene induction, if any, following exposure to ETS (Figure 2). ETS exposure levels averaged 6.3 ± 0.6 mg/m^3^ (mean ± SD). *Gclc* was slightly induced, with *Gclc* levels averaging 110% and 120% of control values at 1 and 2 hr, respectively (*p* < 0.05). Two hours after exposure, *Nqo1* was only increased to 200% of control values (*p* = 0.02, *t*-test).

**Figure 3:**
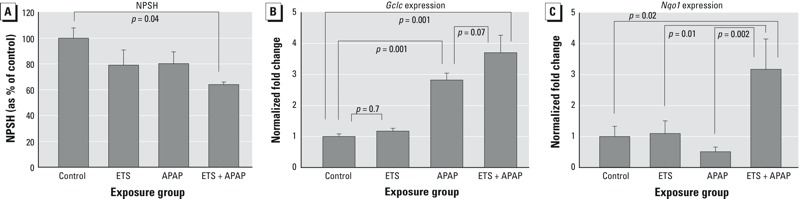
Effects of environmental tobacco smoke (ETS) (5 mg/m^3^), acetaminophen (APAP), and the combination of ETS + APAP on nonprotein sulfhydryl (NPSH) loss and oxidant stress response gene expression. Mice were exposed to ETS for 10 min 1 hr after APAP (or vehicle) administration. NPSH levels were determined in mice euthanized immediately after ETS exposure; gene expression was determined in mice euthanized 2 hr after ETS exposure. (*A*) NPSH levels in nasal respiratory/transitional mucosa in controls and after exposure to APAP, ETS, or APAP + ETS. Data are presented as the mean ± SE and are normalized to total protein. Groups contained 5–7 mice. (*B*) *Gclc* and (*C*) *Nqo1* expression in nasal respiratory/transitional mucosa in controls and after exposure to APAP, ETS, or APAP + ETS. APAP was administered intraperitoneally at a dose of 100 mg/kg; ETS exposures were of 10-min duration at a total particulate concentration of 5 mg/m^3^. *Gclc* expression in the APAP group was significantly higher than in controls (*p *= 0.001) *Nqo1* expression in the APAP groups did not differ from that in controls (*p *= 0.26). Data are presented as the mean fold change ± SE and are normalized to control values. Groups contained 4–6 mice. *p < 0.05 compared with respective control.

To examine the potential for an APAP-ETS interaction, mice were exposed to ETS 1 hr after administering 100 mg/kg APAP because at this time, there was a significant decrease of NPSH (20%) but no induction of antioxidant genes in the RTM (see Figure 1). The dose of 100 mg/kg was selected because at this dose, APAP affected all measures of oxidative stress (see Figure 1). Mice were euthanized immediately after ETS exposure to determine NPSH levels (Figure 3A) and 2 hr after ETS exposure (3 hr after APAP administration) to assess gene expression because both *Gclc* and *Nqo1* would have been induced at that time by ETS (Figure 2). Notably, *Gclc*, but not *Nqo1*, was induced by APAP at this time point (Figure 1).

Exposure to ETS alone and to APAP alone were associated with nonsignificant decreases in NPSH (20% and 19%, respectively), whereas exposure to APAP followed by exposure to ETS caused a 40% reduction in NPSH (*p* = 0.04 compared with control, Figure 3A). NPSH levels were significantly reduced compared with control values in only the APAP + ETS groups. The NPSH levels (81%) in APAP-treated mice in this study were not different from those observed in controls. In contrast, 1 hr after exposure to 100 mg/kg APAP, NPSH levels in RTM were significantly lower than in controls (80% of control values) (Figure 1A). In both experiments, NPSH was measured 1 hr after administering APAP. ETS exposure levels averaged 5.2 ± 0.4 mg/m^3^ (mean ± SD).

*Gclc* expression in the APAP and the APAP + ETS groups was significantly increased over that in controls (Figure 3B). In the ETS group, *Gclc* expression averaged 1.2-fold of control, similar to that in the previous experiment (Figure 2A); however, in this case, a significant difference from the controls was not observed. In the APAP and APAP + ETS groups, *Gclc* expression averaged 2.8- and 3.7-fold of control values, suggesting an additive or greater interaction. The difference in *Gclc* expression in the APAP and APAP + ETS groups approached statistical significance (*p* = 0.07).

*Nqo1* expression was significantly increased compared with controls in only the APAP + ETS group (Figure 3C), and the expression in this group was significantly greater than in either the APAP or the ETS group. In the ETS group, *Nqo1* expression averaged 1.3-fold of control values, a somewhat smaller response than in the previous experiment (Figure 3B); direct comparison of the response in this experiment and that in the preceding one did not reveal a significant difference. *Nqo1* expression was reduced in the APAP group, but the decrease was not significant compared with controls (*p* > 0.9), consistent with the previously observed lack of change in expression for *Nqo1* in RTM 3 hr after administering APAP (see Figure 1C). ETS exposure levels averaged 4.7 ± 0.5 mg/m^3^ (mean ± SD).

**Figure 4:**
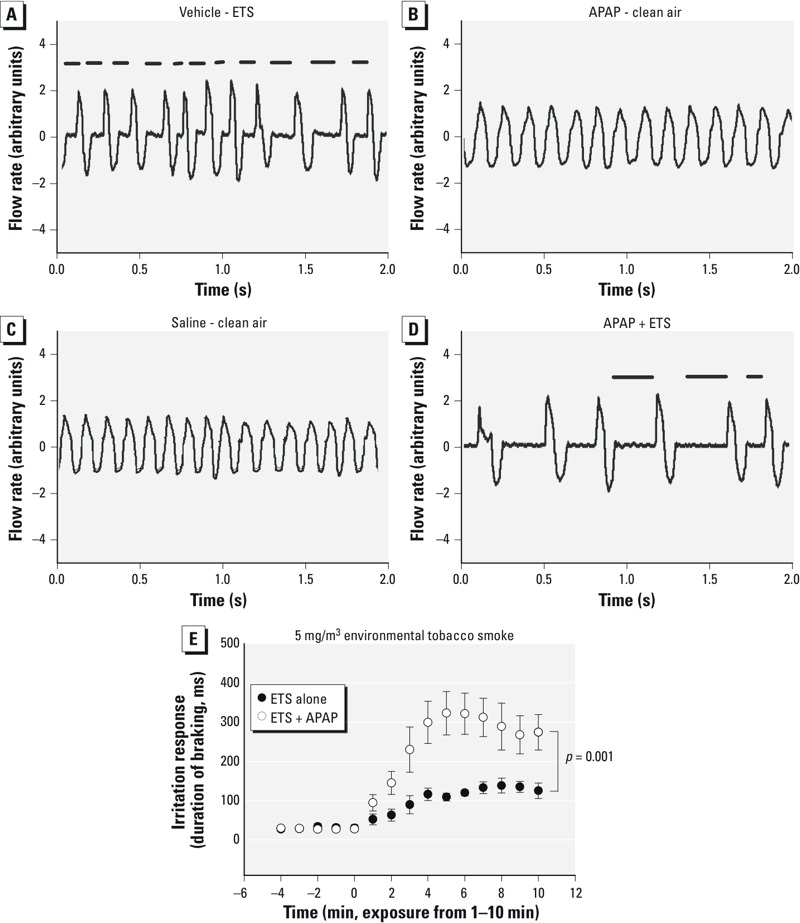
Representative breathing patterns of mice exposed to (*A*) acetaminophen (APAP) vehicle (saline) followed by 5 mg/m^3^ environmental tobacco smoke (ETS), (*B*) 100 mg/kg APAP followed by clean air, (*C*) APAP vehicle (saline) followed by clean air, (*D*) 100 mg/kg APAP followed by ETS. The clean air or ETS exposure occurred 1 hr following vehicle or APAP injection. Plotted are representative respiratory air flow rate patterns (arbitrary units, expiration is up) during these exposures. No braking is observed in control exposures (*C*) or with APAP alone (*B*). Marked braking, indicated by the periods of zero flow (indicated by the horizontal bars above the flow wave lines) was observed in ETS and ETS + APAP–exposed mice. (*E*) Time course of modulation of ETS-induced reflex irritation response by APAP. Data are presented as the mean duration of braking (msec) ± SE. Groups contained 5–10 mice. Data were analyzed by repeated measures analysis of variance (ANOVA); *p*-values shown in the figure represent the *p*-value for the entire 10-min exposure period.

*Irritation reflex.* ETS induces the sensory irritation reflex response as indicated by the induction of braking at the onset of each expiration. This response is illustrated by the period of no airflow (horizontal line) in each breath in Figure 4A; no such response was observed after administration of APAP or vehicle (Figures 4B,C), but an enhanced response was seen in APAP + ETS mice (Figure 4D). This response was quantitated by calculating the 1-min average duration of braking in each animal (Figure 4E). Although APAP alone did not elicit this response, ETS (4.5 ± 0.6 mg/m^3^, mean ± SD) produced a moderate response that was significantly increased in APAP-pretreated mice, indicating that APAP potentiated the irritation response.

**Figure 5:**
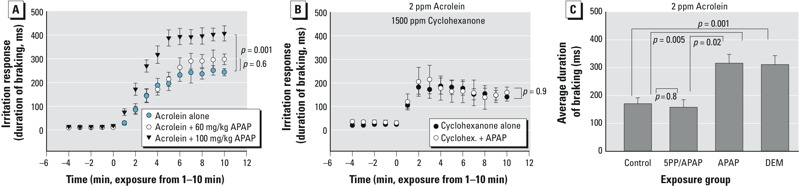
Nasal trigeminal chemosensory nerve reflex irritation responses to (*A*) 2.3 ± 0.4 ppm acrolein (mean ± SD) following vehicle, 60 mg/kg, or 100 mg/kg acetaminophen (APAP), and (*B*) 1,590 ± 130 ppm cyclohexanone following vehicle or 100 mg/kg APAP administration. (*C*) Modulation of the nasal trigeminal chemosensory nerve irritant reflex response to 2.9 ± 0.15 ppm acrolein (control) (mean ± SD) by APAP, 5-phenyl-1pentyne (5-PP) pretreatment followed by APAP, or diethyl maleate (DEM). (*A,B*) Time courses include a 5-min clean air baseline (–5 to 0 min) followed by irritant exposure starting at time 0 (*p*-values are shown in figures). Data are presented as the mean duration of braking (msec) ± SE. Groups contained 5–10 mice. The response to acrolein did not differ in untreated controls, DEM vehicle–treated controls, or 5-PP–treated controls; therefore, these data were all pooled to form the single control group that is shown. Data are presented as the mean duration of braking (msec) ± SE. Data were analyzed by repeated measures analysis of variance (ANOVA) followed by the Newman–Keuls test. *p*-Values shown in the figure represent the *p*-value for the entire 10-min exposure period. Groups contained 4–8 mice.

To investigate the oxidant basis for this interaction, we examined the effects of APAP on the irritation response to the TRPA1-specific oxidant vapor acrolein and to the nonoxidant TRPV1-agonist vapor cyclohexanone (Willis et al. 2011) (Figures 5A,B). APAP had no effects on the response to cyclohexanone, but it potentiated the response to acrolein. To establish a dose–response relationship, two additional dose groups (60 and 200 mg/kg) were included in the acrolein sensory irritation experiment. The response to acrolein was slightly but not significantly (*p* = 0.6) elevated by a 60-mg/kg dose of APAP (Figure 5A), and the response was significantly (*p* = 0.001) elevated at a dose of 100 mg/kg. At a dose of 200 mg/kg, APAP produced braking during the baseline (data not shown). Additional studies were performed to further characterize the role of oxidative stress in the APAP potentiation of the irritant response (Figure 5C). These studies focused on the APAP potentiation of the acrolein irritant response rather than on ETS because acrolein is a single agent known to act through TRPA1 (Willis et al. 2011). As observed previously, the irritation response to acrolein was potentiated by APAP. The potentiation was absent in 5-PP–treated mice. Pretreatment with DEM 1 hr before acrolein exposure potentiated the acrolein response. Nasal RTM NPSH levels in the DEM-treated mice averaged 75 ± 5.4% percent of control values (compared with 100 ± 6.6% in controls, *p* = 0.04, *n* = 4 in each group). This level is similar to that caused by APAP exposure (Figure 1A). Furthermore, 5-PP had no effects on the irritant response to acrolein in mice that were not given APAP, and the DEM vehicle had no effects on the response to acrolein (data not shown). In fact, the durations of braking were within 50 msec of each other and did not differ significantly among any of the control groups (untreated, DEM vehicle–treated and 5-PP–treated); therefore, for the sake of simplicity, these groups were pooled to form the composite control group for this study.

## Discussion

The present study showed that APAP, at supratherapeutic doses, modulated airway oxidative stress responses and respiratory irritant responses to ETS. That adverse respiratory responses to ETS can be enhanced by the commonly used analgesic APAP is a novel concept. More than 97% of children use acetaminophen at recommended doses of ≤ 15 mg/kg before they are 2 years old (Kogan et al. 1994). Historically, ETS exposure in nonsmoking populations has been high. During the period from 1988–1991, > 80% of nonsmoking adults in the United States were passively exposed to ETS (Pirkle et al. 1996). More recently, ETS exposure in nonsmokers has ranged from 52.5% in 1999 to 25.3% in 2012 (Homa et al. 2015). Approximately 30% of children are exposed to ETS in automobiles (King et al. 2012). The ubiquitous use of APAP coupled with the high frequency of exposure to ETS highlights the widespread potential for adverse health effects were a toxicologically significant interaction between APAP and ETS to occur.

Our initial studies indicated that a 60-mg/kg dose of APAP did not cause significant changes in measures of oxidative stress: nasal GSH was not significantly decreased and *Gclc* expression was significantly higher than in controls, but *Nqo1* expression was not. Additionally, 60 mg/kg APAP caused a slight increase in the acrolein response, but the increase was not statistically significant. APAP doses of 100 mg/kg were used for subsequent studies because this was the lowest dose that produced significant changes in all parameters. Future studies could more thoroughly define the effects of APAP at lower doses. Interestingly, at 200 mg/kg, APAP produced a sensory irritation response, suggesting there may be sufficient electrophile produced at this dose to interact with airway sensory nerves. This finding is consistent with the observation that NAPQI can directly activate the TRPA1 channel (Nassini et al. 2010). For humans, the recommended dose of APAP is 15 mg/kg. Therapeutic blood levels of APAPare 5–20 μg/mL, and hepatoxicity is associated with blood levels > 150 μg/mL (Rumack and Matthew 1975) in adults. Although the dosage used herein was higher than the recommended dose, peak blood levels were 35 μg/mL in the adult mice used in this study. This finding is similar to that of Gu et al. (2005), who also reported that blood APAP decreased to 10 μg/mL within 1 hr after a 100-mg/kg dose. Thus, this dosing regimen resulted in near-therapeutic APAP levels that were well below the threshold for overt liver toxicity. The ip route of administration used in the present study may have led to higher peak APAP levels than those that would be observed after oral administration because absorption is slower following oral administration than ip administration; the liver first-pass effect would occur following either dosage regimen.

Female mice were used for these studies. Female mice are less susceptible to APAP hepatotoxicity than males (Sheng et al. 2013) but are more sensitive to the pulmonary toxicity of metabolically activated chemicals such as naphthalene (Van Winkle et al. 2002). Future studies are needed to determine whether sensitivity to the pulmonary effects of APAP and ETS differs between male and female mice.

At a dose of 100 mg/kg, APAP clearly induced oxidative stress in the airways, as indicated by a decrease in NPSH and induction of oxidant stress–response genes. It has previously been shown that APAP depletes nasal NPSH (Gu et al. 2005), but at much higher, hepatotoxic doses. That *Nrf2* ^–/–^ mice demonstrated reduced responsiveness to APAP provides evidence that the oxidant stress gene response was mediated, at least in part, through this transcription factor. The toxic response to APAP is due to metabolic activation via CYP to the strong electrophile NAPQI, and CYP is expressed throughout the respiratory tract of the mouse (Ding and Kaminsky 2003; Hinson et al. 2010). Perhaps local activation of APAP is involved in the responses observed herein. The present results do not, however, rule out the possibility of hepatic events (escape of activated APAP, decrease of blood GSH) as a contributing factor to the oxidative stress response observed in this study (Gu et al. 2005; Phimister et al. 2004). Because 5-PP inhibits CYP metabolism systemically, these data do not provide insight into the exact role of hepatic versus local nasal activation of APAP.

ETS contains thousands of chemicals, many of which are oxidants (Gilmour et al. 2006). Exposure to 5 mg/m^3^ ETS for 10 min caused a modest oxidative stress response as indicated by the slight induction of an oxidative stress gene response. Although responses of similar magnitude were observed in the first ETS experiment (Figure 2) and in the ETS + APAP experiment (Figure 3), the responses were significantly different from controls in only one experiment. This finding suggests that the ETS exposure represented a threshold response level because the magnitude of response was quite low, and the response was significantly different from the control in only one of two experiments. If so, this response may be of concern because the exposure level is similar to that obtained in a car containing an active smoker (Sendzik et al. 2009), and the duration of the exposure was only 10 min. Future studies should determine if slightly higher concentrations of ETS or longer ETS exposure time results in a repeatedly observed oxidative stress response.

The interaction between APAP and ETS is likely due to the oxidant properties of both agents. The NPSH loss response of both agents appeared to be additive based on the 40% decrease in NPSH observed in the APAP + ETS groups compared with the 20% decrease in NPSH observed in the APAP-only and ETS-only groups, although no formal statistical test was performed to confirm this possibility. The induction of *Gclc* also appeared to be additive, although the differences between APAP and APAP + ETS did not quite attain statistical significance. The *Nqo1* response clearly indicated a synergistic interaction between APAP and ETS; neither agent alone produced a response, but the combination caused a clear induction. A synergistic interaction was also observed with respect to the reflex irritation response to ETS. APAP alone did not cause reflex irritation, but it markedly increased the irritation response to ETS.

Our observation that APAP enhanced the irritation response to ETS suggests that APAP can alter complex integrated airway responses. ETS stimulates chemosensory nerves via the oxidant-sensitive TRPA1 receptor (Andrè et al. 2008). The present study confirms an oxidant basis for the interaction of APAP with ETS. APAP potentiated the irritant response to the oxidant acrolein, which is a known TRPA1 agonist. That APAP had no effect on the nonoxidant TRPV1 agonist cyclohexanone suggests that the effects of APAP are oxidant-specific. Treatment with 5-PP blocked the modulation of acrolein irritation, indicating that oxidant-induced sensory irritation was caused by generation of a metabolite, likely NAPQI, rather than by a pharmacological effect of the parent APAP. APAP is known to be activated by nasal CYP, and the nasal toxicity of APAP is independent of liver activation (Gu et al. 2005). At a dose that produced a similar level of GSH loss to that produced by APAP, DEM mimicked APAP’s effects on acrolein irritation, suggesting that GSH loss may contribute to the interaction. DEM has been shown to potentiate the nasal toxicity of another metabolically activated toxicant, naphthalene (Phimister et al. 2004). Perhaps nasal GSH acts to detoxify acrolein, and loss of GSH enhances acrolein penetration to chemosensory nerve endings that innervate the nasal mucosa. Overall, these findings provide evidence of an oxidant basis for the effect of APAP on respiratory responses; they also suggest that tissue antioxidant levels may be a determinant of sensitivity to inhaled irritant chemicals.

The public health impacts of ETS have been well studied. ETS exposure is associated with increased asthma incidence and severity (Coogan et al. 2015; IOM 2000; Kanchongkittiphon et al. 2015). Multiple epidemiological studies in both adults and children have revealed an association between increased APAP use since 1980 and the increased prevalence of asthma since that time (Barr et al. 2004; Beasley et al. 2011; Etminan et al. 2009). These findings have led to the “APAP hypothesis”; that is, acetaminophen may contribute to asthma causation, perhaps through its oxidant properties (McBride 2011). This hypothesis is based simply on the observation that both APAP use and asthma prevalence have increased since 1980; however, it is controversial because of the potential for confounding in epidemiological studies and because of the lack of experimental evidence (Heintze and Petersen 2013; Holgate 2011). Moreover, any factor that has changed since 1980, such as air pollution, environmental chemical exposure, and others, may be responsible for the increase in asthma prevalence. The results of the present study lend credence to the APAP hypothesis by supporting a novel and biologically plausible mechanism whereby APAP may contribute to the development of asthma, specifically by enhancing the effects of other asthma risk factors such as ETS. However, the present study only provides information about single short-term exposures to APAP and ETS. Future studies are needed to address the effects of repeated exposures to these agents and to investigate potential differences in outcome if ETS exposure preceded APAP administration. Nevertheless, oxidative stress is thought to play a role in asthma pathogenesis (Holguin 2013; Riedl and Nel 2008), and the present study indicates an oxidative stress–based interaction between environmentally relevant levels of ETS and supratherapeutic levels of APAP.

## Conclusions

Our findings indicate that APAP administered to mice at supratherapeutic levels acts as an airway oxidant and potentiates acute airway responses to environmentally relevant levels of another airway oxidant, ETS. These results suggest that APAP may exert adverse effects on the respiratory tract; however, in the absence of confirmatory evidence from human studies, it is premature to suggest, even tentatively, changes in clinical practice.

## References

[r1] Alarie Y (1973). Sensory irritation by airborne chemicals.. CRC Crit Rev Toxicol.

[r2] Alarie Y (1981). Bioassay for evaluating the potency of airborne sensory irritants and predicting acceptable levels of exposure in man.. Food Cosmet Toxicol.

[r3] Aleksunes LM, Slitt AL, Maher JM, Dieter MZ, Knight TR, Goedken M (2006). Nuclear factor-E2-related factor 2 expression in liver is critical for induction of NAD(P)H:quinone oxidoreductase 1 during cholestasis.. Cell Stress Chaperones.

[r4] Andrè E Campi B Materazzi S Trevisani M Amadesi S Massi D 2008 Cigarette smoke-induced neurogenic inflammation is mediated by αβ-unsaturated aldehydes and the TRPA1 receptor in rodents. J Clin Invest 118 7 2574 2582, doi:10.1172/JCI34886 18568077 PMC2430498

[r5] Barr RG Wentowski CC Curhan GC Somers SC Stampfer MJ Schwartz J 2004 Prospective study of acetaminophen use and newly diagnosed asthma among women. Am J Respir Crit Care Med 169 7 836 841, doi:10.1164/rccm.200304-596OC 14711794

[r6] Bataille AM Manautou JE 2012 Nrf2: a potential target for new therapeutics in liver disease. Clin Pharmacol Ther 92 3 340 348, doi:10.1038/clpt.2012.110 22871994 PMC3704160

[r7] Bautista DM Jordt S Nikai T Tsuruda PR Read AJ Poblete J 2006 TRPA1 mediates the inflammatory actions of environmental irritants and proalgesic agents. Cell 124 6 1269 1282, doi:10.1016/j.cell.2006.02.023 16564016

[r8] Beasley RW Clayton TO Crane J Lai CK Montefort SR von Mutius E 2011 Acetaminophen use and risk of asthma, rhinoconjunctivitis, and eczema in adolescents: International Study of Asthma and Allergies in Childhood phase three. Am J Respir Crit Care Med 183 2 171 178, doi:10.1164/rccm.201005-0757OC 20709817

[r9] Boyland E, Chasseaud LF (1967). Enzyme-catalysed conjugations of glutathione and unsaturated compounds.. Biochem J..

[r10] Caceres AI Brackmann M Elia MD Bessac BF del Camino D D’Amours M 2009 A sensory neuronal ion channel essential for airway inflammation and hyperreactivity in asthma. Proc Natl Acad Sci USA 106 22 9099 9104, doi:10.1073/pnas.0900591106 19458046 PMC2684498

[r11] Chan JY, Kwong M 2000 Impaired expression of glutathione synthetic enzyme genes in mice with targeted deletion of the Nrf2 basic-leucine zipper protein. Biochim Biophys Acta 1517 1 19 26, doi:10.1016/S0167-4781(00)00238-4 11118612

[r12] Cichocki JA Smith GJ Mendoza R Buckpitt AR Van Winkle LS Morris JB 2014a Sex differences in the acute nasal antioxidant/antielectrophilic response of the rat to inhaled naphthalene. Toxicol Sci 139 1 234 244, doi:10.1093/toxsci/kfu031 24563378

[r13] Cichocki JA Smith GJ Morris JB 2014b Tissue sensitivity of the rat upper and lower extrapulmonary airways to the inhaled electrophilic air pollutants diacetyl and acrolein. Toxicol Sci 142 1 126 136, doi:10.1093/toxsci/kfu165 25145656

[r14] Coogan PF Castro-Webb N Yu J O’Connor GT Palmer JR Rosenberg L 2015 Active and passive smoking and the incidence of asthma in the Black Women’s Health Study. Am J Respir Crit Care Med 191 2 168 176, doi:10.1164/rccm.201406-1108OC 25387276 PMC4347433

[r15] Cruzan G Bus J Hotchkiss J Harkema J Banton M Sarang S 2012 CYP2F2-generated metabolites, not styrene oxide, are a key event mediating the mode of action of styrene-induced mouse lung tumors. Regul Toxicol Pharmacol 62 1 214 220, doi:10.1016/j.yrtph.2011.10.007 22041433

[r16] Ding X Kaminsky LS 2003 Human extrahepatic cytochromes P450: function in xenobiotic metabolism and tissue-selective chemical toxicity in the respiratory and gastrointestinal tracts. Annu Rev Pharmacol Toxicol 43 149 173, doi:10.1146/annurev.pharmtox.43.100901.140251 12171978

[r17] Etminan M Sadatsafavi M Jafari S Doyle-Waters M Aminzadeh K Fitzgerald JM 2009 Acetaminophen use and the risk of asthma in children and adults: a systematic review and metaanalysis. Chest 136 5 1316 1323, doi:10.1378/chest.09-0865 19696122

[r18] Gilmour MI Jaakkola MS London SJ Nel AE Rogers CA 2006 How exposure to environmental tobacco smoke, outdoor air pollutants, and increased pollen burdens influences the incidence of asthma. Environ Health Perspect 114 627 633, doi:10.1289/ehp.8380 16581557 PMC1440792

[r19] Gloede E Cichocki JA Baldino JB Morris JB 2011 A validated hybrid computational fluid dynamics-physiologically based pharmacokinetic model for respiratory tract vapor absorption in the human and rat and its application to inhalation dosimetry of diacetyl. Toxicol Sci 123 1 231 246, doi:10.1093/toxsci/kfr165 21705714

[r20] Gu J, Cui H, Behr M, Zhang L, Zhang QY, Yang W (2005). In vivo mechanisms of tissue-selective drug toxicity: effects of liver-specific knockout of the NADPH-cytochrome P450 reductase gene on acetaminophen toxicity in kidney, lung, and nasal mucosa.. Mol Pharmacol.

[r21] Ha MA Smith GJ Cichocki JA Fan L Liu YS Caceres AI 2015 Menthol attenuates respiratory irritation and elevates blood cotinine in cigarette smoke exposed mice. PLoS One 10 2 e0117128, doi:10.1371/journal.pone.0117128 25679525 PMC4334501

[r22] Hart SG, Cartun RW, Wyand DS, Khairallah EA, Cohen SD (1995). Immunohistochemical localization of acetaminophen in target tissues of the CD-1 mouse: correspondence of covalent binding with toxicity.. Fundam Appl Toxicol.

[r23] Heintze K Petersen KU 2013 The case of drug causation of childhood asthma: antibiotics and paracetamol. Eur J Clin Pharmacol 69 6 1197 1209, doi:10.1007/s00228-012-1463-7 23292157 PMC3651816

[r24] Hinson JA Roberts DW James LP 2010 Mechanisms of acetaminophen-induced liver necrosis. Handb Exp Pharmacol 196 369 405, doi:10.1007/978-3-642-00663-0_12 20020268 PMC2836803

[r25] Holgate ST 2011 The acetaminophen enigma in asthma. Am J Respir Crit Care Med 183 2 147 148, doi:10.1164/rccm.201007-1135ED 21242591

[r26] Holguin F 2013 Oxidative stress in airway diseases. Ann Am Thorac Soc 10 suppl S150 S157, doi:10.1513/AnnalsATS.201305-116AW 24313766

[r27] Homa DM, Neff LJ, King BA, Caraballo RS, Bunnell RE, Babb SD (2015). Vital signs: disparities in nonsmokers’ exposure to secondhand smoke—United States, 1999–2012.. MMWR Morb Mortal Wkly Rep.

[r28] IOM [Institute of Medicine (US) Committee on the Assessment of Asthma and Indoor Air] (2000). Clearing the Air: Asthma and Indoor Air Exposures..

[r29] Jaeschke H McGill MR Ramachandran A 2012 Oxidant stress, mitochondria, and cell death mechanisms in drug-induced liver injury: lessons learned from acetaminophen hepatotoxicity. Drug Metab Rev 44 1 88 106, doi:10.3109/03602532.2011.602688 22229890 PMC5319847

[r30] Kanchongkittiphon W Mendell MJ Gaffin JM Wang G Phipatanakul W 2015 Indoor environmental exposures and exacerbation of asthma: an update to the 2000 review by the Institute of Medicine. Environ Health Perspect 123 6 20, doi:10.1289/ehp.1307922 25303775 PMC4286274

[r31] King BA Dube SR Tynan MA 2012 Secondhand smoke exposure in cars among middle and high school students—United States, 2000–2009. Pediatrics 129 3 446 452, doi:10.1542/peds.2011-2307 22311992 PMC4583774

[r32] Klaassen CD Reisman SA 2010 Nrf2 the rescue: effects of the antioxidative/electrophilic response on the liver. Toxicol Appl Pharmacol 244 1 57 65, doi:10.1016/j.taap.2010.01.013 20122946 PMC2860427

[r33] Kogan MD Pappas G Yu SM Kotelchuck M 1994 Over-the-counter medication use among US preschool-age children. JAMA 272 13 1025 1030, doi:10.1001/jama.1994.03520130063034 8089884

[r34] Larkin EK Gao YT Gebretsadik T Hartman TJ Wu P Wen W 2015 New risk factors for adult-onset incident asthma. A nested case-control study of host antioxidant defense. Am J Respir Crit Care Med 191 1 45 53, doi:10.1164/rccm.201405-0948OC 25408961 PMC4299629

[r35] Lin MC, Wang EJ, Patten C, Lee MJ, Xiao F, Reuhl KR (1996). Protective effect of diallyl sulfone against acetaminophen-induced hepatotoxicity in mice.. J Biochem Toxicol.

[r36] Livak KJ Schmittgen TD 2001 Analysis of relative gene expression data using real-time quantitative PCR and the 2^–ΔΔC(T)^ method. Methods 25 4 402 408, doi:10.1006/meth.2001.1262 11846609

[r37] Lowry OH, Rosebrough NJ, Farr AL, Randall RJ (1951). Protein measurement with the Folin phenol reagent.. J Biol Chem.

[r38] McBride JT 2011 The association of acetaminophen and asthma prevalence and severity. Pediatrics 128 6 1181 1185, doi:10.1542/peds.2011-1106 22065272

[r39] McGill MR Jaeschke H 2013 Metabolism and disposition of acetaminophen: recent advances in relation to hepatotoxicity and diagnosis. Pharm Res 30 9 2174 2187, doi:10.1007/s11095-013-1007-6 23462933 PMC3709007

[r40] Morris JB 2012 Biologically-based modeling insights in inhaled vapor absorption and dosimetry. Pharmacol Ther 136 3 401 413, doi:10.1016/j.pharmthera.2012.08.017 22964085

[r41] Morris JB 2013 Nasal dosimetry of inspired naphthalene vapor in the male and female B6C3F1 mouse. Toxicology 309 66 72, doi:10.1016/j.tox.2013.04.009 23619605

[r42] Morris JB Symanowicz PT Olsen JE Thrall RS Cloutier MM Hubbard AK 2003 Immediate sensory nerve-mediated respiratory responses to irritants in healthy and allergic airway-diseased mice. J Appl Physiol (1985) 94 4 1563 1571, doi:10.1152/japplphysiol.00572.2002 12626476

[r43] Nassini R Materazzi S Andrè E Sartiani L Aldini G Trevisani M 2010 Acetaminophen, via its reactive metabolite *N*-acetyl-*p*-benzo-quinoneimine and transient receptor potential ankyrin-1 stimulation, causes neurogenic inflammation in the airways and other tissues in rodents. FASEB J 24 12 4904 4916, doi:10.1096/fj.10-162438 20720158

[r44] Nel A Xia T Mädler L Li N 2006 Toxic potential of materials at the nanolevel. Science 311 5761 622 627, doi:10.1126/science.1114397 16456071

[r45] Phimister AJ Lee MG Morin D Buckpitt AR Plopper CG 2004 Glutathione depletion is a major determinant of inhaled naphthalene respiratory toxicity and naphthalene metabolism in mice. Toxicol Sci 82 1 268 278, doi:10.1093/toxsci/kfh258 15319489

[r46] Pirkle JL, Flegal KM, Bernert JT, Brody DJ, Etzel RA, Maurer KR (1996). Exposure of the US population to environmental tobacco smoke: the Third National Health and Nutrition Examination Survey, 1988 to 1991.. JAMA.

[r47] Riedl MA Nel AE 2008 Importance of oxidative stress in the pathogenesis and treatment of asthma. Curr Opin Allergy Clin Immunol 8 1 49 56, doi:10.1097/ACI.0b013e3282f3d913 18188018

[r48] Roberts ES Alworth WL Hollenberg PF 1998 Mechanism-based inactivation of cytochromes P450 2E1 and 2B1 by 5-phenyl-1-pentyne. Arch Biochem Biophys 354 2 295 302, doi:10.1006/abbi.1998.0679 9637739

[r49] Rumack BH, Matthew H (1975). Acetaminophen poisoning and toxicity.. Pediatrics.

[r50] Saunders CJ Li WY Patel TD Muday JA Silver WL 2013 Dissecting the role of TRPV1 in detecting multiple trigeminal irritants in three behavioral assays for sensory irritation. F1000Res 2 74, doi:10.12688/f1000research.2-74.v1 24358880 PMC3814916

[r51] Sedlak J, Lindsay RH (1968). Estimation of total, protein-bound, and nonprotein sulfhydryl groups in tissue with Ellman’s reagent.. Anal Biochem.

[r52] Sendzik T Fong GT Travers MJ Hyland A 2009 An experimental investigation of tobacco smoke pollution in cars. Nicotine Tob Res 11 6 627 634, doi:10.1093/ntr/ntp019 19351785 PMC2688598

[r53] Sheng Y Liang Q Deng Z Ji L Wang Z 2013 Acetaminophen induced gender-dependent liver injury and the involvement of GCL and GPx. Drug Discov Ther 7 2 78 83, doi:10.5582/ddt.2013.v7.2.78 23715506

[r54] Spiess PC Morin D Williams CR Buckpitt AR 2010 Protein thiol oxidation in murine airway epithelial cells in response to naphthalene or diethyl maleate. Am J Respir Cell Mol Biol 43 3 316 325, doi:10.1165/rcmb.2009-0135OC 19843705 PMC2933547

[r55] Van Winkle LS Gunderson AD Shimizu JA Baker GL Brown CD 2002 Gender differences in naphthalene metabolism and naphthalene-induced acute lung injury. Am J Physiol Lung Cell Mol Physiol 282 5 L1122 L1134, doi:10.1152/ajplung.00309.2001 11943679

[r56] Vijayaraghavan R, Schaper M, Thompson R, Stock MF, Alarie Y (1993). Characteristic modifications of the breathing pattern of mice to evaluate the effects of airborne chemicals on the respiratory tract.. Arch Toxicol.

[r57] Willis DN Liu B Ha MA Jordt SE Morris JB 2011 Menthol attenuates respiratory irritation responses to multiple cigarette smoke irritants. FASEB J 25 12 4434 4444, doi:10.1096/fj.11-188383 21903934 PMC3236628

